# From image biomarkers to treatment decisions: multimodal AI and clinical translation in gynecologic oncology

**DOI:** 10.3389/fonc.2026.1960963

**Published:** 2026-10-09

**Authors:** Mohamad Bakir, Mohammad Khaldoun Al Masri, Yousef Mushtaha, Narmeen Shaikh, Mohammed Raddaoui, Khalid Said Mohammad

**Affiliations:** 1 Department of Medicine, College of Medicine, Alfaisal University, Riyadh, Saudi Arabia; 2 Department of Biostatistics, Epidemiology and Public Health, College of Medicine, Alfaisal University, Riyadh, Saudi Arabia; 3 Department of Anatomy, College of Medicine, Alfaisal University, Riyadh, Saudi Arabia

**Keywords:** artificial intelligence, clinical decision support, gynecologic oncology, multimodal AI, precision oncology, radiomics

## Abstract

Artificial intelligence (AI) is rapidly expanding across gynecologic oncology, but most applications remain focused on image interpretation and retrospective prediction rather than direct support of treatment decisions. This review examines how AI is progressing from imaging biomarkers toward multimodal decision-support systems that integrate radiologic, pathologic, molecular, and clinical information. We summarize current applications in ovarian, endometrial, and cervical cancers, with particular attention to preoperative risk stratification, surgical planning, radiotherapy, systemic treatment selection, treatment-response prediction, and multidisciplinary decision-making. Multimodal approaches are especially promising because they reflect the way clinical decisions are made and may provide greater value than models based on a single data source. However, the current evidence remains dominated by retrospective and often single-center studies, with limited external and prospective validation. Data heterogeneity, domain shift, incomplete multimodal datasets, bias, workflow integration, regulatory requirements, and model monitoring remain important barriers to routine implementation. We therefore outline a stepwise pathway for clinical translation, from robust multicenter validation and prospective human–AI evaluation to clinical-impact studies and post-deployment monitoring. The next phase of AI in gynecologic oncology should prioritize clinically meaningful decisions and measurable patient or workflow benefit rather than incremental gains in predictive performance alone.

## 1 Introduction

### 1.1 The central role of AI in gynecologic oncology

Multimodal AI in gynecologic oncology typically develops separate models for imaging, digital pathology, and molecular/clinical data, then integrates their outputs to improve the accuracy of prognostic and predictive tools. These integrated models can more effectively stratify preoperative risk, predict actionable molecular subtypes from pictures, and facilitate customized decisions for surgery, systemic therapy, and surveillance. Nevertheless, most systems are still under development, and comprehensive multicenter validation is required before standard clinical application.

#### 1.1.1 Evolution of advanced imaging and molecular classification

In high-grade serous ovarian cancer (HGSOC), distinct machine-learning models are developed utilizing CT radiomics (e.g., omental texture), histopathological characteristics (e.g., tumor nuclear size), and clinicogenomic variables, subsequently amalgamated within a late-fusion multimodal framework; n = 444 patients with predominantly advanced high-grade serous ovarian cancer treated at a single tertiary center; retrospective, with internal cross-validation and no external cohort; this synthesis enhanced risk stratification for progression-free and overall survival beyond clinicogenomic factors alone (1).

#### 1.1.2 Biological complexity and technical challenges in gynecologic AI

Artificial intelligence is increasingly used for imaging, pathology, genomics, and clinical decision support in gynecologic cancers, but biological complexity and fragmented data constrain translation to routine care. Across ovarian, endometrial, and cervical cancers, clinical translation is limited by inter- and intratumoral heterogeneity, uncertain generalizability across settings, and the difficulty of integrating multimodal data within evolving guideline-based treatment pathways. Gynecologic malignancies exhibit significant heterogeneity in histology, molecular subtype, and clinical behavior, particularly ovarian cancer, complicating diagnosis, therapy, and prognosis (2, 3). Evolving molecular classifications in endometrial and cervical cancer introduce complexity for AI models that must adapt to shifting risk categories and treatment guidelines (3, 4). Scarcity of large, prospective, and diverse cohorts, together with data privacy and bias concerns, impedes the development of effective multimodal models and may exacerbate inequities (5, 6).

### 1.2 Limitations of prior reviews and current literature gaps

Single-modality AI, including radiomics and imaging-based deep learning, has shown promising diagnostic and predictive performance. However, most studies remain retrospective, heterogeneous, and poorly externally validated, limiting their generalizability and clinical utility. This is particularly evident in gynecologic ultrasound studies, where incomplete reporting of patient selection, scanner characteristics, and preprocessing contributes to a high risk of bias.

In gynecologic radiomics, the majority of research is single-center, retrospective, and utilizes tiny datasets, resulting in overfitting and limited generalizability (7). In endometrial cancer radiomics, the evidence is deemed inadequate, with just a small fraction of studies meeting established quality criteria (8).

Radiomics pipelines exhibit variation in acquisition, segmentation, feature extraction, and reporting, complicating cross-study comparisons and the establishment of standards (7). Reviews highlight persistent challenges regarding feature stability, reproducibility, and model generalizability, categorizing radiomics as a discipline still in a “developmental phase (9).” Standardization and harmonization across scanners and institutions are recognized as essential prerequisites for routine clinical implementation (10, 11).

Current ultrasound and radiomics AI applications in gynecologic oncology mostly focus on ovarian mass diagnosis, with limited models available for endometrial, cervical, and myometrial malignancies, as well as for predicting treatment response and survival outcomes (12, 13). Despite strong AUCs for ovarian tumor classification, existing clinical tools (e.g., ADNEX) continue to surpass numerous AI models, raising doubts about their additional value in decision-making (13). Reviews on ovarian cancer AI emphasize that, while aggregated performance is favorable, heterogeneity, bias, and insufficient explainability hinder clinical implementation and personalized therapy approaches (14).Single-modality models overlook the whole clinical and biological context; multimodal AI is emphasized as a means to enhance robustness and accuracy (15).

Numerous gynecologic AI models are developed using single-center or localized/national datasets, resulting in spectrum bias and restricting applicability to wider populations (16, 17). In the domain of ultrasound AI for pelvic malignancies, 95% of studies exhibited a significant risk of bias, frequently lacking appropriate train/test splits and calibration; only five incorporated external validation (13). Data privacy, anonymization, and administration are complex and labor-intensive, especially given the substantial numbers required for AI training. Clinical integration necessitates significant IT and computational infrastructure, along with resilient data pipelines, which exert pressure on current systems (18). In gynecologic computational pathology, non-standardized metrics and evolving disease classifications impede comparison and translation (19). Numerous prediction models lack well-defined implementation strategies, stakeholder involvement, or post-deployment evaluation (20). The opaque nature of deep learning undermines trust; explainable models and visualization techniques in gynecologic digital pathology are still developing (21). Automation bias and insufficient transparency are identified as significant clinical issues in gynecologic oncology (22). Limited physician training, resource limitations, and workflow interruptions (e.g., inadequate EHR integration, increased workload) impede adoption in gynecologic oncology (23).

### 1.3 Rationale, novelty, and aim of this review

A comprehensive systematic review of post-diagnostic AI in oncology found that most oncological AI studies remain experimental, lack prospective clinical validation or implementation, and often do not show a significant effect on clinical outcomes (24). A systematic evaluation in gynecologic tumors revealed 71 AI research, with 90% utilizing fewer than 1000 cases and frequently without external validation, hindering quantitative synthesis and restricting clinical applicability (25). In digital pathology for gynecologic malignancies, internal assessments often show high performance; however, the lack of external validation and varied methodologies limit extensive multicenter application (19). A partial exception is a multicenter retrospective model that diagnoses ovarian cancer from routine laboratory tests; it achieved an AUC of 0.949 in an internal validation set (n = 3,007) and 0.882 and 0.884 in two external hospital cohorts (n = 5,641 and n = 2,344). All three settings were retrospective, and the model has not been evaluated prospectively or in deployment (26). Two studies have compared large language model outputs against gynecologic tumor board decisions. In a single-center retrospective comparison across 599 consecutive patients with cervical, endometrial, ovarian, or vulvar cancer, ChatGPT 5.0 prompted to follow ESGO guidance agreed with the multidisciplinary team on FIGO stage in 77.0% of cases, with discordance in treatment recommendations of 8.2% for chemotherapy and 6.8% for targeted therapy; staging disagreement was highest in early-stage endometrial cancer (32.6%), and discrepancies in surgical and systemic recommendations were most pronounced in recurrent ovarian and cervical disease (4). A smaller feasibility study evaluated three language models against physician assessment on five fictitious case vignettes and found broad agreement, with divergence concentrated in the more complex cases (27). Both studies are retrospective or hypothetical, both present the model with written case summaries rather than the primary imaging, pathology, and molecular data, and both measure concordance with team decisions rather than patient outcomes. They establish that current models reproduce guideline-concordant staging and management in straightforward cases and that agreement falls where multimodal integration and individualized judgment are required; they do not establish clinical safety or benefit (4, 27). In cancer, major obstacles to adoption include skewed or heterogeneous data, inadequate clinical validation, workflow and user-design issues, and inconsistent reporting (28, 29).

Recent reviews have comprehensively examined radiomics, ultrasound-based AI, digital pathology, radiogenomics, and the broader application of AI across gynecologic malignancies. However, these reviews have largely organized the literature according to imaging modality, cancer type, or predictive task. The unresolved translational question is different: when does an AI-generated prediction become sufficiently validated and clinically actionable to influence a specific treatment or management decision? The present review therefore adopts a decision-centered framework linking model output to clinical action, comparator, validation stage, workflow integration, and potential patient-level consequence. Rather than cataloging AI applications alone, we examine the transition from diagnostic and prognostic biomarkers toward multimodal decision-support systems that directly inform clinical care.

### 1.4 Literature search and review approach

Literature search and review approach. This article is a narrative review focused on the clinical translation of artificial intelligence in gynecologic oncology. For the revision, we identified relevant literature through targeted searches of PubMed/MEDLINE and Scopus through August 2026, supplemented by citation tracking of relevant primary studies and recent reviews. Search concepts combined terms related to artificial intelligence, machine learning, deep learning, radiomics, multimodal AI, gynecologic oncology, ovarian cancer, endometrial cancer, cervical cancer, surgery, radiotherapy, treatment response, molecular biomarkers, and clinical decision support. We placed particular emphasis on primary studies providing external, multicenter, prospective, or clinically oriented validation, along with recent systematic reviews and relevant methodological or reporting frameworks. Because the aim was to provide a clinically oriented narrative synthesis rather than an exhaustive systematic review or meta-analysis, we did not conduct formal PRISMA-based study selection or study-level risk-of-bias scoring.

## 2 Conceptual framework: AI modalities and scope

### 2.1 Definitions and scope of AI technologies

To clarify the scope, we classify AI technologies into five categories. Unimodal AI refers to models relying on a single data source, such as isolated imaging or digital pathology. Multimodal imaging AI incorporates multiple imaging sequences or modalities, such as combining MRI with PET/CT. Cross-domain multimodal AI extends this by integrating imaging with distinct data types, including pathology, molecular profiles, and clinical records. Clinical decision-support AI shifts from descriptive outputs to predictions explicitly linked to actionable clinical interventions. Finally, treatment-effect models attempt to estimate the differential benefit a patient may receive from alternative treatments, moving beyond baseline prognostic prediction toward causal inference.

Traditional radiomics, deep learning techniques, multimodal integration models, and clinical decision-support systems differentiate diagnostic artificial intelligence from therapeutic guidance AI. In gynecologic oncology, radiomics, deep learning, and fusion models serve as decision-support mechanisms: diagnostic AI emphasizes detection and characterization, while treatment-oriented AI focuses on risk assessment, response evaluation, and therapy planning. AI applications in gynecologic oncology are based on quantitative imaging (radiomics), advanced image features (deep learning), and the integration of these with clinical and genomic information (multimodal fusion). They support decision-making from diagnosis to prognosis and treatment selection, though this varies by task and data range.

Radiomics refers to extracting quantitative imaging characteristics (shape, intensity, texture) after acquisition and segmentation, then selecting and modeling these characteristics to forecast clinical outcomes such as diagnosis, staging, prognosis, or therapy response (9, 12, 30). Deep learning employs neural networks (e.g., CNNs) to extract characteristics directly from images, facilitating tasks including tumor detection, segmentation, classification, and outcome prediction in gynecological cancers (21, 31). Fusion models integrate handcrafted radiomic features, learned imaging representations derived from deep neural networks, and/or clinical variables. In locally advanced cervical cancer, CerviPro amalgamates pre- and post-treatment CT scans, extracted radiomic features, and clinical data to forecast disease-free survival, with multimodal fusion surpassing single-category models and categorizing patients into high- and low-risk groups for tailored treatment (32).

AI radiomics in gynecological oncology is extensively employed for tumor identification and characterization (e.g., stage, histological subtype, lymph node metastases, LVSI, myometrial invasion) (7). Ultrasound AI models assist in the preoperative assessment of ovarian masses and the forecasting of histological subtypes to facilitate surgical planning in the absence of professional evaluators (12). Digital pathology deep learning algorithms categorize histopathological subtypes and identify gynecological malignancies from whole-slide images (21). Radiomics and deep learning are employed for forecasting and treatment prediction, encompassing responses to chemotherapy/radiotherapy and survival outcomes (7, 31). Ultrasound AI is anticipated to forecast molecular characteristics and therapeutic responses (such as POLE, PD L1, treatment resistance, and recurrence) to enhance treatment personalization (12).

### 2.2 Multimodal data streams in gynecologic oncology

Artificial intelligence is increasingly used to integrate ultrasound, MRI, CT, and PET/CT in gynecologic oncology; however, integrating multiple modalities remains in its early, largely preclinical stages. Artificial intelligence in gynecologic oncology primarily uses imaging techniques to derive quantitative “radiomics” and deep learning features for diagnosis, staging, and prognosis, with early efforts to integrate imaging and clinical information. Genuine cross-modal integration (e.g., ultrasound combined with MRI and PET/CT) is infrequent, and extensive clinical implementation is still limited.

Reviews characterize AI and radiomics as a “digital biopsy,” employing MRI, CT, and PET to predict molecular changes like HRD in ovarian cancer or MSI in endometrial cancer, connecting imaging phenotypes with genotypes to inform precision treatment (3). Deep learning analyses highlight that utilizing radiological and pathological imagery enhances AI’s capabilities in tumor detection, segmentation, classification, necrosis evaluation, and prognosis forecasting in both primary and metastatic gynecological cancers (31, 33). Radiogenomics analyses underscore issues related to data constraints, model applicability, and ethical or regulatory challenges, particularly in the integration of imaging with omics and clinical information (3, 10).

### 2.3 Clinical endpoints of interest

#### 2.3.1 Tumor classification, staging, and preoperative assessment

Artificial intelligence in gynecologic oncology is used throughout the diagnostic continuum to categorize tumors, predict histologic or molecular classifications, determine staging, and assess surgical resectability using multimodal imaging and pathological information. Deep learning applied to radiological and pathological images improves tumor classification and histologic subtype identification, facilitating tumor detection, segmentation, and necrosis assessment in primary and metastatic gynecologic cancers and markedly increasing baseline diagnostic precision (21, 31). AI radiomics effectively forecasts staging, lymphovascular space invasion (LVSI), and deep myometrial invasion across MRI, CT, PET, and ultrasound for stage and risk stratification, facilitating risk-oriented management (2, 7). This is also illustrated in the evaluation of nodal metastasis, where multimodal deep learning-radiomics-clinical models preoperatively forecast pelvic lymph node metastasis in early cervical cancer, yielding AUCs near 0.8 to support personalized lymphadenectomy choices (34, 35). In contrast, direct evidence for predicting explicit surgical resectability is scarce; existing models primarily assess operability indirectly through staging, nodal status, and risk stratification metrics rather than using specific resectability scoring systems (22).

#### 2.3.2 Treatment response, recurrence, and survival outcomes

AI models in gynecologic cancers show moderate to high predictive efficacy for neoadjuvant therapy response, long-term recurrence, and overall survival in research contexts, but insufficient toxicity forecasting and prospective validation impede clinical application. A systematic review of 13 studies on radiomics and machine learning, encompassing 5,113 patients with advanced ovarian cancer, reported a median AUC of approximately 0.77 for forecasting response to neoadjuvant chemotherapy (NACT) and 0.82 for predicting optimal or complete cytoreduction, alongside a median overall accuracy of 73% (36). In locally advanced cervical cancer, MRI-derived radiomics and machine learning achieved AUCs of approximately 0.82 to 0.86, with 74-75% accuracy in differentiating complete from incomplete responses to neoadjuvant chemotherapy or chemoradiotherapy (37, 38). A two-center deep-learning radiomics nomogram for predicting response to neoadjuvant chemotherapy reported high discriminatory performance, with AUCs ranging from approximately 0.91 to 0.96 across the development, internal-validation, and external-validation datasets. However, these estimates should be interpreted in the context of a relatively small external cohort, class imbalance in the development data that required synthetic minority oversampling, geographic restriction of the cohort, and the absence of prospective validation. The results are therefore encouraging but should not be interpreted as establishing clinical readiness (39). Moreover, integrating imaging and molecular data in high-grade serous ovarian cancer markedly improves prediction of NACT response relative to clinical data alone (RECIST AUC 0.78 versus 0.47) (40). A comprehensive review of radiomics identified 27 studies highlighting the strong potential of AI to predict recurrence, metastasis, and survival, while acknowledging considerable heterogeneity in methods and their predominance in single-center research (7). Extensive cervical cancer radiomics and multi-omics models consistently achieve elevated AUCs (often exceeding 0.8–0.9) for predicting 1- to 5-year disease-free survival and overall survival to categorize patient risk, although they remain limited to research applications (2, 32). Ultimately, although assessments indicate that AI models have theoretical potential for forecasting treatment-associated toxicities, validated, toxicity-specific models are not yet firmly established in gynecologic populations, and existing evidence remains in its preliminary, conceptual phase (2, 41). In general, AI models show commendable to exceptional differentiation for neoadjuvant response and survival or recurrence in retrospective contexts, often surpassing independent clinical models; however, they still require extensive prospective, multicenter validation. These performance estimates should not be interpreted as direct head-to-head comparisons because the studies differ substantially in cohort composition, outcome prevalence, modeling strategy, validation design, and treatment setting; several also lack external validation or complete calibration reporting. **Figure 1** summarizes the overall pathway from multimodal data acquisition and AI processing to clinical decision support and downstream clinical impact.

**Figure 1:**
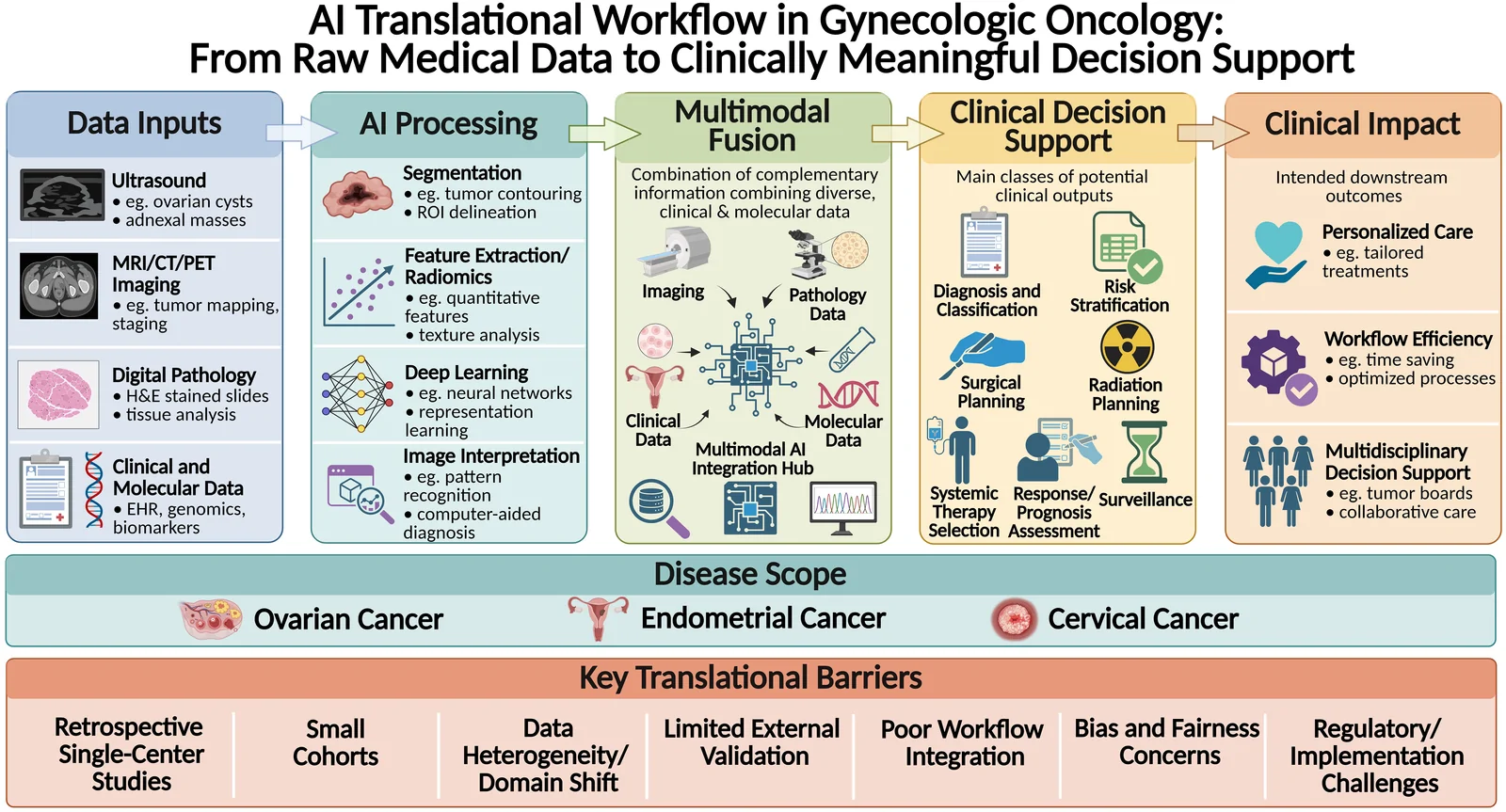
Translational workflow of multimodal AI in gynecologic oncology. Imaging, digital pathology, and clinical and molecular data can be processed using segmentation, radiomics, deep learning, and image interpretation approaches, and subsequently integrated via multimodal AI. These systems may support diagnosis, risk stratification, surgical and radiation planning, selection of systemic therapy, response and prognostic assessment, and surveillance across ovarian, endometrial, and cervical cancers. Major barriers to clinical translation include data heterogeneity, limited external validation, workflow integration, bias, and regulatory and implementation challenges. Created in BioRender. Helal, B. (2026) https://BioRender.com/hypncyr.

## 3 Current landscape of AI in gynecologic oncology by disease and use case

AI in gynecologic oncology now spans ultrasound, MRI, CT, PET, digital pathology, molecular testing, and routine clinical variables. The central question is no longer whether a model separates malignant from benign findings, but rather if it changes staging, surgical triage, radiation planning, systemic treatment choice, or surveillance in a reproducible way (7, 22, 42, 43).

The current evidence base also carries a repeated translation problem. Many models report high discrimination, yet few use locked algorithms, prespecified thresholds, calibration plots, decision curve analysis, external cohorts, prospective workflow testing, or patient-centered endpoints. A deployable model in this field must connect a prediction to a clinical action. It must also show that the action is safe across scanners, hospitals, operators, tumor subtypes, and patient groups.

### 3.1 Ovarian cancer

Most ovarian cancer AI work begins with adnexal mass classification. Ultrasound models now include handcrafted radiomics, convolutional neural networks, and tools linked to O-RADS, IOTA Simple Rules, and ADNEX (12, 44–47). These systems support triage, referral to gynecologic oncology, and reduction of operator variation. The deployment issue is setting. Expert ultrasound already performs well, so AI adds the most value in non-expert practice, low-volume sites, and lesions in intermediate-risk groups. In these settings, AI needs calibration against O-RADS and IOTA outputs, clear referral thresholds, and prospective testing in routine scans.

Preoperative risk stratification is more relevant to treatment selection. CT, MRI, and PET models have been trained to predict histotype, tumor burden, peritoneal spread, nodal disease, residual disease after debulking, and response to neoadjuvant chemotherapy (48, 49). These outputs can support the choice between primary debulking and neoadjuvant chemotherapy; yet the evidence is not mature enough to replace laparoscopy, surgical judgment, or multidisciplinary review. Imaging alone misses several clinical and patient-level factors like frailty, nutrition, ascites control, center volume, anesthetic risk, and surgeon skill. Multimodal models that combine imaging with tumor markers such as CA-125 and HE4, inflammatory markers, BRCA status, HRD status, and pathology may offer greater treatment value than image-only classifiers (1, 50–52).

AI/radiogenomic models may predict platinum response, BRCA/HRD-associated phenotypes and outcome risk, but these imaging-derived surrogates should complement rather than substitute validated molecular testing used for PARP-inhibitor selection (1, 50–52).

### 3.2 Endometrial cancer

Endometrial cancer is the clearest example of AI moving from imaging biomarker to preoperative pathway design. MRI radiomics and deep learning models have been studied for deep myometrial invasion, cervical stromal invasion, lymphovascular space invasion (LVSI), lymph node metastasis, tumor grade, histotype, and molecular subtype (53–58). These variables guide sentinel node mapping, lymphadenectomy, surgical route, and adjuvant therapy intensity. MRI-based models are also being developed for microsatellite instability (MSI), PD-L1 expression, and p53-abnormal disease (59–62). This aligns AI with the molecular risk structure now used in modern endometrial cancer care.

For treatment planning, the most useful model is not a standalone image classifier. It is a preoperative risk model that combines MRI features, biopsy grade, histology, LVSI probability, molecular subtype, age, body mass index, comorbidity, and planned nodal assessment. This form of AI can separate patients suitable for minimally invasive surgery from patients needing a higher-risk pathway. It can also support adjuvant radiation and systemic therapy intensity after tumor board review. The evidence remains limited by small cohorts, manual segmentation, variable MRI protocols, and few prospective workflow studies (53, 54, 63). Prognostic models for recurrence and survival are promising, but they need surveillance-linked endpoints and proof that altered follow-up improves detection or reduces harm (57, 58).

Endometrial cancer also shows why model reporting must move beyond the area under the curve. A model that predicts deep invasion or LVSI needs a stated decision threshold that changes nodal staging, operating time, and adjuvant therapy. Similarly, a model that predicts molecular class needs clear linkage to POLE-mutated, mismatch repair-deficient, no specific molecular profile, or p53-abnormal pathways. Without that linkage, the model remains an imaging exercise rather than a treatment tool.

### 3.3 Cervical cancer

In cervical cancer, AI has developed across staging, local disease characterization, chemoradiation response, and radiation oncology tasks. MRI and PET radiomics have been used to predict tumor extent, nodal disease, parametrial invasion, treatment response, residual disease, early relapse, and survival (64–66). This is clinically relevant, as treatment depends on local extent, nodal burden, and expected response to chemoradiation. Multi-scanner and multicenter PET/MRI work provides stronger evidence than single-site models, but many studies still lack prospective testing of treatment impact (66, 67).

Radiation oncology is the most mature technical area in the context of cervical cancer management, where deep learning models support target delineation, organ-at-risk segmentation, brachytherapy contours, dose prediction, treatment plan optimization, and adaptive replanning (68–74). The best-supported use is contouring support, which reduces planning workload and variation, while dose optimization and adaptive radiotherapy remain closer to technical proof of concept. The overall clinical value depends on plan quality, review burden, failure detection, toxicity endpoints, and safe handover between software and radiation oncologists.

Treatment response prediction also needs sharper clinical framing. Routine treatment change without trial evidence is not justified; therefore, a model that predicts poor chemoradiation response should lead to a trial of dose escalation, altered brachytherapy planning, closer assessment of residual disease, or structured salvage review. Models for early relapse risk are useful only with a surveillance plan that changes imaging frequency, examination timing, or trial referral.

### 3.4 Less-studied gynecologic malignancies

Vulvar cancer, vaginal cancer, and uterine sarcomas remain underrepresented in the literature. Rare tumors pose a central AI challenge: small datasets increase overfitting, restrict external validation, and limit subgroup testing. These cancers also lack large public imaging datasets and uniform treatment pathways. Future work needs multicenter rare tumor registries, shared imaging definitions, pathology review, and federated learning. For uterine sarcoma, MRI AI has a clear diagnostic role in distinguishing sarcoma from leiomyoma, but the treatment-planning evidence remains limited (75).

Rare tumors also test fairness and transportability. A model built from common cancers cannot be assumed to be valid for uncommon tumor types. Differences in anatomy, imaging protocols, surgical margins, nodal mapping, radiation fields, and systemic treatment options change what each predictor means. As such, deployment should start with registry-linked decision support, not autonomous recommendations.

The relative maturity, emerging decision-support applications, and principal evidence gaps across the three major gynecologic malignancies are summarized in **Figure 2**.

**Figure 2:**
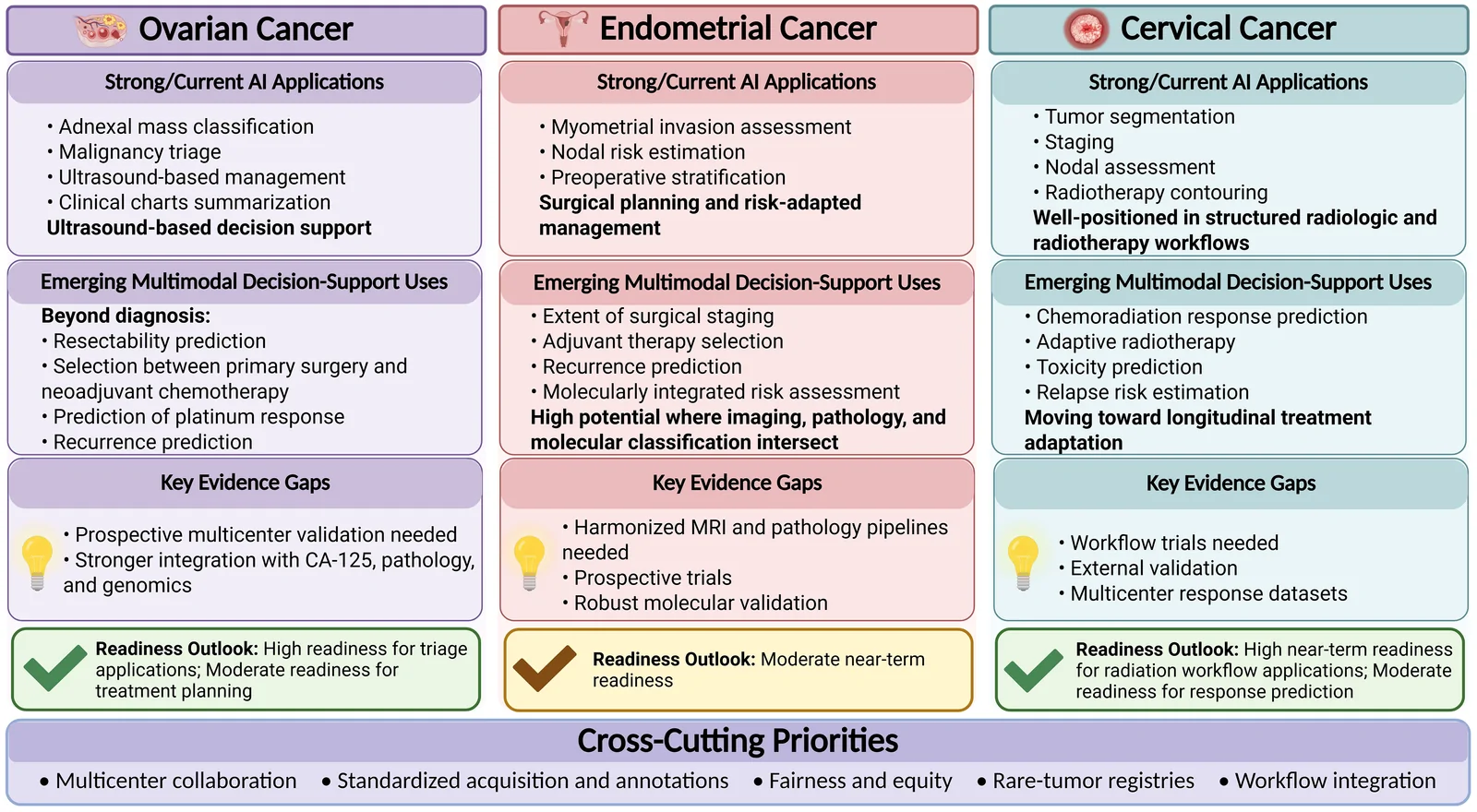
Disease-specific landscape and clinical readiness of AI in major gynecologic cancers. The image summarizes established and emerging AI applications for ovarian, endometrial, and cervical cancer, along with the key evidence gaps that limit clinical adoption. Current strengths differ by disease, with greater maturity in ultrasound-based ovarian tumor assessment, preoperative risk stratification in endometrial cancer, and structured radiology and radiotherapy workflows in cervical cancer. Cross-cutting priorities include multicenter validation, standardized data acquisition and annotation, equitable model development, rare-tumor registries, and workflow integration. Created in BioRender. Helal, B. (2026) https://BioRender.com/hypncyr.

## 4 From imaging biomarker to treatment decision

### 4.1 AI for surgical planning

AI for surgery remains less mature than diagnostic AI. The most relevant attempts involve ovarian cancer resectability, residual disease, operative complexity, peritoneal spread, and postoperative risk (1, 40, 48, 49). Endometrial models address nodal risk and depth of invasion, which shape the surgical route and nodal plan (53–58). These tasks can be challenging, as surgical outcomes depend on biology, anatomy, patient fitness, team expertise, and institutional resources. A deployable surgical AI tool needs to report predicted benefit, predicted harm, and the reason for recommending primary surgery, neoadjuvant therapy, minimally invasive surgery, or open surgery.

Surgical deployment also needs outputs that fit the operating pathway. A useful report should identify likely disease sites, expected complexity, the risk of incomplete cytoreduction, the need for upper abdominal expertise, and the likelihood of conversion. For robotic or minimally invasive surgery, the model should support case selection rather than promote a surgical route. The true benchmark is not image accuracy, but whether AI improves triage, reduces futile laparotomies and unplanned conversions, and enables timely referral to high-volume teams.

### 4.2 AI for radiation therapy planning

Radiation planning has the clearest clinical path for AI-guided treatment decisions. Segmentation models reduce contouring time and interobserver variation, particularly for target and organ-at-risk segmentation in cervical cancer. Dose prediction and adaptive radiotherapy can improve consistency, but they require strict quality assurance and real-time failure checks (68–74). Toxicity prediction is less mature since many toxicity models use retrospective dose-volume and clinical data with uneven outcome capture. Deployment needs prospective monitoring of plan edits, contour corrections, dose metrics, acute toxicity, late toxicity, and treatment delays.

Distinguish between technical utility and clinical utility. A contouring model that saves time but creates hidden target misses is unsafe. Meanwhile, a dose model that improves conformity but increases late toxicity has limited value. Radiation AI should be evaluated with clinician correction rates, dosimetric pass rates, treatment start delays, toxicity, and patient-reported outcomes; local commissioning should be part of this process before routine use.

### 4.3 AI for systemic therapy selection

Among treatment-related applications, systemic therapy selection remains the least developed and least clinically validated area. Ovarian cancer models for platinum sensitivity, BRCA status, HRD-related imaging patterns, and neoadjuvant chemotherapy response offer the clearest route (1, 50–52). Endometrial models for MSI, p53-abnormal disease, and PD-L1 expression suggest a bridge to immunotherapy or targeted therapy decisions (59–61). Cervical cancer models predicting response to chemoradiation may help guide escalation trials, but they are not yet ready to support routine treatment changes (64–66).

The field should avoid using imaging surrogates as substitutes for validated biomarkers. Radiogenomics, for instance, can be valuable in settings with limited tissue availability or spatial heterogeneity, but it should complement pathology and molecular testing. A useful systemic therapy model should clearly define the treatment decision, the comparator strategy, and the outcome it is expected to change. Without this structure, it cannot meaningfully guide therapy selection.

We need to clearly distinguish between predicting a patient’s prognosis and predicting the actual effect of a specific treatment. Just because a model accurately forecasts recurrence or platinum sensitivity doesn’t mean it can tell us if an alternative therapy would work better. To safely guide clinical decisions, models have to weigh counterfactuals answering “what if” scenarios using data from prospective randomized trials or rigorous causal inference. While advanced statistical methods can help handle observational data, they are still highly vulnerable to confounding and selection bias. Because of this, current gynecologic AI models are best viewed as risk-stratification tools, not definitive guides for prescribing treatments.

A central distinction in this review is between prognostic prediction and treatment-effect prediction. A prognostic model estimates a patient’s outcome under the care she is expected to receive: it answers what is likely to happen. A treatment-effect model estimates how that outcome would differ under one treatment compared with an alternative: it answers what should be done. A model that predicts recurrence or platinum resistance with high discrimination therefore carries no information about whether a different regimen would serve that patient better, because the counterfactual outcome under the alternative treatment is never observed.

Estimating that quantity requires either randomization or an explicit causal-inference design. Target-trial emulation makes the design assumptions visible by specifying the eligibility criteria, treatment strategies, assignment procedure, follow-up period, and outcome of the hypothetical randomized trial that the observational analysis is intended to emulate. The estimand must be a conditional average or heterogeneous treatment effect rather than an outcome risk, since the clinical question is who benefits differentially rather than who does badly. Doubly robust estimators, which remain consistent if either the outcome model or the treatment-assignment model is correctly specified, offer some protection against misspecification. Time-varying confounding and treatment switching require explicit handling and are particularly relevant in gynecologic oncology because response assessment, interval debulking, and maintenance decisions occur sequentially, so that a variable that predicts the next treatment is often also affected by the previous one.

Even with these methods, confounding by indication and selection bias remain because clinicians rarely fully record the reasons they chose one treatment over another. Current gynecologic AI models are therefore best understood as risk-stratification tools. To our knowledge, none has been shown to identify differential treatment benefit, and prospective validation against a prespecified treatment decision remains necessary before using any such model to allocate therapy.

### 4.4 AI in multidisciplinary tumor board support

The most realistic near-term deployment is human-in-the-loop tumor board support. AI can integrate imaging, pathology, molecular data, labs, comorbidity, and guideline logic to flag risk groups and suggest guideline-concordant options. Concordance is easier in early and straightforward disease; however, recurrent and advanced disease needs human review due to prior treatments, trial access, organ function, symptoms, patient goals, and uncertainty. Therefore, readiness should be judged with reporting and risk-of-bias tools, including TRIPOD+AI, PROBAST+AI, CLAIM, DECIDE-AI, and FUTURE-AI (76–80). Regulation and adoption of such models also need audit trails, model monitoring, local validation, cybersecurity, equity testing, and clear accountability (81, 82). Overall, AI in gynecologic oncology is ready to support triage and treatment planning in selected settings, but it is not yet ready to make autonomous treatment decisions.

More advanced decision-support systems may evolve toward tool-using or multi-agent architectures that retrieve external evidence, query structured clinical data, and simulate specialist roles. These systems introduce failure modes beyond conventional predictive modeling, including incorrect evidence retrieval, citation errors, hallucinated recommendations, inappropriate tool selection, loss of longitudinal context, and failure to recover safely from erroneous intermediate steps. For such systems, auditability and explicit clinician override are therefore at least as important as recommendation concordance. These approaches remain in the early stages and should not be conflated with clinically validated tumor-board decision support.

## 5 Multimodal AI as the real next step

Artificial intelligence in gynecologic oncology is now moving beyond isolated image interpretation toward multimodal systems that combine imaging with clinical, pathologic, and molecular information. This shift matters because clinicians do not make treatment decisions from a single data source, and single-modality models cannot capture the biological heterogeneity, treatment context, and patient-specific factors that shape outcomes. Multimodal AI therefore represents the most credible next step for the field, with the potential to improve risk stratification, predict treatment response more accurately, and support more personalized decision-making across ovarian, endometrial, and cervical cancers.

### 5.1 Why single-modality models are insufficient

A single modality captures only one facet of a tumor, so a model confined to it is blind to information that can change management. An MRI shows anatomy but not molecular subtype; histology shows cell type but not the disease’s extent across the abdomen; serum markers and treatment history sit entirely outside imaging. Tumor complexity cannot be inferred from a single data source (3, 83) yet most current systems still rely on just one. Oncology data are heterogeneous by nature, spanning imaging, genomics, pathology, records, and outcomes (84). The gap is sharpest in gynecologic cancers, where variation between and within tumors drives prognosis and treatment resistance that no single view resolves (3, 85). This is why most current single-modality systems, however accurate on their narrow task, cannot support personalized treatment planning (15, 86): the limiting factor is not the algorithm but the fraction of the clinical picture they can see. The field is therefore moving away from isolated prediction tasks and toward integrated models that can support diagnosis, biomarker discovery, prognosis, and treatment-response prediction within a single patient-level framework. The evidence shows this: across a range of cancer prediction tasks, multimodal models have outperformed the best single-source models (87, 88).

Multimodality should not be regarded as intrinsically superior for every clinical task. When the target is narrowly defined and strongly represented within a single data source, for example automated organ-at-risk segmentation in radiotherapy, morphological classification on pathology, or standardized adnexal-mass characterization on high-quality ultrasound, a unimodal model may be sufficient and may be easier to validate, deploy, and maintain. Additional modalities are most valuable when they contribute nonredundant information that materially changes the clinical decision. The relevant comparison is therefore not multimodal versus unimodal complexity per se, but whether additional data improve calibration, clinical utility, transportability, or decision-making beyond an appropriate simpler comparator.

### 5.2 Imaging + clinical variables

The first practical step beyond imaging alone is often combining imaging with clinical variables. Clinical data provides information that scans do not directly encode, including laboratory markers, demographics, medications, family history, and other structured record elements. Because different modalities encode complementary information, multimodal input is often advantageous rather than merely optional (88).

In ovarian cancer, the strongest primary evidence for combining imaging with clinical variables comes from ultrasound-radiomics studies of epithelial tumor subtyping. A study combined ultrasound radiomic features with clinical variables (age, CA125, and CA19-9) to predict type I versus type II epithelial ovarian cancer and performed almost identically in the training cohort (AUC 0.83) and a held-out internal test cohort (AUC 0.82) (89). The similar performance in the training and held-out internal test cohorts indicated no large apparent decrement in discrimination within that source population; however, internal testing cannot exclude overfitting, data leakage, center-specific confounding, or limited transportability, which require genuinely independent external validation. Tang et al. extended this approach by adding menopausal state, ascites, CA125, SCC, HE4, and ROMA indices; their comprehensive model outperformed the radiomics-only model, achieving a test-set AUC of 0.886 versus 0.731, with a statistically significant difference and favorable calibration and clinical-practicability analyses (90). A separate ovarian mass study by Chiappa et al. supports the same multimodal principle for malignancy-risk prediction, combining transvaginal ultrasound radiomics with CA125 and menopausal status, and achieving 91% accuracy in an independent held-out test set of 35 patients (91). Reviews of ovarian radiomics describe this pattern as recurring: multimodal models that integrate imaging with clinical variables tend to improve subtype-specific diagnosis, but the field still needs multicenter prospective validation and better standardization before those gains can be considered clinically established (85).

### 5.3 Imaging + pathology + molecular features

A more advanced form of multimodal integration links imaging with pathology and genomics. Radiogenomics aims to infer molecular phenotype from imaging features, thereby bridging radiologic appearance and tumor biology noninvasively (3, 92). In gynecologic oncology, this approach has been framed as a digital biopsy because it seeks to decode actionable biology from routine imaging without relying solely on tissue sampling (3). This is clinically attractive because tissue biopsy samples only part of a heterogeneous tumor, whereas imaging can assess the whole lesion and metastatic distribution repeatedly over time. Reviews in ovarian cancer describe radiomics and radiogenomics as useful for distinguishing benign from malignant tumors, classifying subtypes, and predicting survival, treatment outcome, metastasis, and recurrence (85, 92). Reviews across cancers also report that integrated imaging- and histopathology-genomic frameworks improve prediction of patient outcomes and molecular subtype identification relative to unimodal systems (93). The biological promise extends beyond prognosis. AI-based radiogenomics in gynecologic cancers is already being discussed in relation to HRD in ovarian cancer, MSI and PI3K activation in endometrial cancer, and HPV-related molecular changes in cervical cancer (3).

In practice, however, this ambition remains largely a proof of concept in gynecologic cohorts. In ovarian cancer, the PROBE study built a radiogenomic model from transvaginal ultrasound images to predict germline BRCA1/2 status in women with morphologically normal ovaries. n = 255 women referred for germline BRCA1/2 testing at a single center between 2013 and 2017; retrospective, with a 75%/25% training–testing split and no external validation; accuracy on the held-out set was modest (0.54–0.64), and the authors framed the work as hypothesis-generating (94). The clinical rationale is nonetheless strong, because BRCA1/2 mutation and the broader homologous-recombination-deficient (HRD) phenotype define eligibility for PARP-inhibitor maintenance, so a reliable imaging surrogate could route patients toward molecular testing earlier.

Beyond radiogenomics, deep learning applied directly to digital pathology (hematoxylin-and-eosin whole-slide images) has advanced furthest in endometrial cancer. The interpretable im4MEC model performed multiclass classification of endometrial cancers into the four molecular categories, POLE-mutated, mismatch-repair deficient, p53-abnormal, and no specific molecular profile, from hematoxylin-and-eosin whole-slide images. n = 2,028 patients with intermediate-to-high-risk disease pooled from the PORTEC-1, PORTEC-2 and PORTEC-3 randomized trials and additional clinical cohorts, with PORTEC-3, n = 393, held out as an independent test set; macro-average AUROC 0.876 on that held-out set, with class-wise values of 0.849 for POLE-mutated, 0.844 for mismatch-repair deficient, 0.883 for no specific molecular profile, and 0.928 for p53-abnormal disease; retrospective, with no prospective evaluation reported (95). Because MMRd status can influence eligibility for immune checkpoint inhibitors, morphology-based molecular prediction could identify patients who warrant confirmatory molecular testing, thereby linking routine pathology to the treatment-selection pathway without replacing validated biomarker ascertainment. Throughout this review, interpret imaging- or morphology-derived molecular predictions primarily as surrogate biomarkers or triage tools that may identify patients for formal molecular testing; they do not establish molecular status or treatment eligibility unless validated against and incorporated with the corresponding clinical assay.

True tri-modal integration of radiomics, pathomics, and genomics remains largely pan-cancer rather than gynecology-specific: interpretable histopathology-genomic fusion improved risk stratification over unimodal models in 9 of 14 cancer types in a pan-cancer analysis (96), whereas gynecologic-specific tri-modal cohorts are only beginning to emerge. Several constraints temper near-term translation. Paired imaging-genomic datasets are scarce, creating a persistent data bottleneck (3); the spatial mismatch between an imaging voxel (a 3-D image unit) and the biopsied tissue complicates ground-truth labeling; and most current evidence is retrospective and single-center.

Large pathology foundation models are shifting the field by learning from massive datasets of whole-slide images, molecular data, and text. This is a major advantage for tasks like biomarker prediction because it drastically reduces the amount of labeled gynecologic data we need. But these massive datasets come with a catch: hidden data overlap, patient duplication, and benchmark contamination. Because of these blind spots, we can’t just assume a broad pan-cancer model is automatically ready for the clinic. Any gynecologic application still demands rigorous, disease-specific external validation.

### 5.4 Practical multimodal architectures

Multimodal systems are usually built with early, intermediate, or late fusion strategies (93, 97). Early fusion aggregates features into one shared representation and remains common because it is comparatively simple to implement. Intermediate fusion uses modality-specific encoders with shared layers to learn cross-modal dependencies and is generally the most expressive approach. Late fusion combines outputs from modality-specific models and is more modular and robust when modalities arrive asynchronously or are missing. These categories map onto the gynecologic work discussed above: the ultrasound radiomics-score-plus-clinical-variable nomograms used for ovarian tumor subtyping effectively perform intermediate-to-late fusion, combining separately derived radiomic and clinical predictors rather than learning a single joint representation from raw inputs.

Contemporary multimodal architectures increasingly extend this classical taxonomy through cross-attention between modality-specific representations, token-based fusion, mixture-of-experts routing, contrastive representation alignment, modality dropout, and masked-modality pretraining. These approaches may improve information sharing and robustness when modalities are incomplete. Importantly, missingness is not always random; complete-case multimodal cohorts may preferentially select patients who receive more intensive diagnostic workup, thereby introducing selection bias. Architecture design should therefore consider both computational missingness and the clinical processes that determine why a modality is absent.

The main architectural constraint is not elegance but missingness. Multimodal medical data are routinely incomplete, and many machine learning methods still assume full data availability (15, 98). Missing modalities reduce usable sample size during training and complicate deployment because models built on complete data often cannot use patients with partial records (15). This is not a niche issue: multimodal healthcare datasets are widely characterized by different structures and missing values, making missing-data handling a central design problem (99).

Recent work suggests two broad solutions. One is imputation or modality synthesis, though this risks altering the original data distribution (99, 100). The other is to design models that handle missingness directly. In comparative evaluation, direct modeling preserved underlying data structure better than imputation and maintained clinical patterns more effectively (100). Transformer-based fusion with modality-specific tokens also improved prediction while accommodating patients with missing modalities, showing that portability depends partly on architectures designed for real clinical incompleteness rather than ideal research datasets (98).

Future architectures are likely to rely more on foundation models, self-supervised pretraining, and federated learning. Reviews in gynecologic radiogenomics argue that foundation models may help overcome labeled-data scarcity through few-shot adaptation, while federated learning offers a privacy-preserving route to larger and more diverse training datasets (3). Similar perspectives from other multimodal clinical domains argue that multicentric datasets and federated infrastructures may enable stronger intermediate-fusion architectures and multimodal foundation models in practice (97, 101).

### 5.5 Clinical promise versus current reality

Multimodal AI has clear clinical promise, but the current evidence base is still stronger for retrospective performance gains than for demonstrated improvements in real patient care. In gynecologic oncology, AI already shows efficacy in tumor characterization, risk stratification, response prediction, radiotherapy planning, and prognostic modeling. The handful of systems tested beyond retrospective datasets illustrate both sides of this gap. Within gynecologic oncology, the ovarian decision-support system described above reached 91% accuracy on an independent held-out test set of 35 patients drawn from a consecutive series of 274 women, with reproducibility assessed across examiners and ultrasound machines (91). The sample is small and the design retrospective, but the multi-examiner, multi-machine testing is closer to real-world conditions than most work in this area. The ovarian decision-support system described above reached 91% accuracy in a prospective cohort. Cautionary cases are equally instructive: a deep-learning system for automated visual evaluation of the cervix initially reported near-perfect accuracy for detecting precancer (102), but this performance proved non-portable and was later attributed to overfitting that internal validation had not exposed, showing how retrospective accuracy can collapse on external data (103).

That promise has not yet translated into routine deployment. Many proposed gynecologic oncology systems still lack robust external validation, and few have demonstrated sustained performance or measurable benefit in real-world use. Across oncology, only a small fraction of AI models have been properly validated, and even fewer have become regulated tools for routine practice (104). Understanding why so few of these models cross the gap from retrospective promise to real-world benefit is therefore the pressing question.

## 6 Why high-performing AI models still fail to translate

Despite rapid advances, AI remains only partly integrated into routine gynecologic oncology. The main challenge is no longer model performance in retrospective studies, but whether these tools remain reliable, interpretable, equitable, and clinically useful across real-world settings. A persistent gap remains between promising technical results and readiness for treatment planning, multicenter use, and workflow integration, largely because of limited validation and implementation.

### 6.1 Data heterogeneity and domain shift

One basic problem is that the inputs to these models are not stable biological measurements. Radiomic features, in particular, are partly artifacts of how an image is acquired: changing the scanner, field strength, reconstruction kernel, or slice thickness can alter the feature values extracted from the same tumor, and a substantial fraction of features prove non-reproducible under such variation (85). A model trained at one institution may therefore be learning that institution’s imaging pipeline as much as its patients’ disease. The same problem extends beyond radiomics to multimodal and routine clinical data, which differ across sites in structure, acquisition protocols, and standards and are further complicated by missingness, variable granularity, and inconsistent representation of clinical concepts (84, 97). Even within a single institution, data are often not research-ready and may be stored under different patient identifiers across systems (86). The result is domain shift: a model trained on one data distribution meets another at deployment and degrades, often beyond what center-specific confounders alone would predict (86).

Two solutions exist, neither of them complete. Image-level standardization harmonizes acquisition and processing before features are extracted; the Image Biomarker Standardization Initiative formalized this approach, reaching consensus reference values for a standardized set of radiomic features together with a common processing scheme and reporting guidelines (105). Feature-level correction instead removes site effects after extraction, most commonly with ComBat, a method originally developed for batch effects in genomic data and since adapted to the multicenter, multi-scanner data typical of gynecologic imaging, including a locally advanced cervical cancer cohort combining MRI and PET across three centers, where it improved feature consistency and cross-center model performance (106, 107). ComBat estimate’s location and scale differences associated with predefined batch variables such as scanner or institution and adjusts feature distributions to reduce those systematic effects while attempting to preserve biological variation. Its limitations include dependence on correct batch specifications, the possibility of removing biologically meaningful variation when biology and site are correlated, and reduced reliability when batches are small or poorly represented. Harmonization helps, but it cannot recover information that has already been lost, and it makes its own assumptions about what counts as a batch.

Some sources of variability cannot be corrected after the fact. Ultrasound is operator-dependent, so the image itself varies with the operator, and inconsistent tumor segmentation and annotation add further noise upstream of any model. Multimodal models also tend to be developed on specific cohorts or geographic settings that do not capture the full variability of practice and patient populations, which limits how far their performance can be expected to transfer (84). Heterogeneity cannot be fully engineered away; it is a built-in feature of medical imaging and clinical data, and any claim of generalizability must be tested against it.

### 6.2 Study design weaknesses

Most AI studies in gynecologic oncology are still built on retrospective, single-center datasets that are small and often unrepresentative. The cases are usually whatever a large referral hospital had available, so the patient mix rarely matches the wider population a model would eventually serve. The problem is sharper for multimodal work, where the number of patients with fully matched imaging, pathology, genomics, and outcomes is far smaller than the number available for any single modality; this scarcity limits not only model performance but also benchmarking and biomarker development (15, 86). The outcome of interest, malignancy, nodal spread, or recurrence, is also usually the rarer class, and that imbalance is often handled by resampling in ways that flatter the model while degrading its calibration.

These are not only questions of sample size but of who is in the sample. When particular subgroups are underrepresented, their outcomes tend to be predicted less accurately, so fairness has to be checked directly rather than assumed; benchmark studies outside gynecology have already shown clear class-imbalance and fairness problems for Black and publicly insured patients (108). The scale of the design problem has been measured. A systematic review of machine-learning prognostic models across oncology found that 84% were at high risk of bias on PROBAST, most often because of too few patients and reliance on splitting a single dataset rather than testing on a new one (109). Radiomics reviews reach the same verdict using the Radiomics Quality Score (110), with oncologic radiomics studies averaging only about a quarter of the available points (111).

A model built on a few hundred selected cases from one hospital, tested only on part of that same data and reported without calibration, will almost always look better than it really is, which is why reporting standards such as PROBAST and the TRIPOD+AI guideline now exist to make these weaknesses visible (76, 112). Weak study design, more than weak algorithms, is the main reason results that look strong on paper so often fail on new data.

### 6.3 Validation weaknesses

The hardest test of a model is not how it performs on a held-out slice of its own dataset but how it performs somewhere else entirely. Good results on an internal split show only that a model is consistent within one dataset; whether it has learned real biology rather than the habits of one center becomes clear only when it is tried on data from a different hospital, scanner, or time period. By that standard, the gynecologic literature is thin. In a systematic review of ovarian-mass radiomics, fewer than one study in five carried out any external validation (113); a comparable neuro-oncology review found that under a third did (111); and even among externally tested oncology models, about half remained at high risk of bias (109). Prospective evaluation is rarer still, and testing on patients treated later than the development cohort, the least demanding check against drift over time, or across countries is largely absent from disease-specific gynecologic work, which leaves the real clinical impact of most models unknown (85).

This matters because pooling data across institutions is one of the clearest routes to generalizable models. A recent international study trained ultrasound models on 17,119 images from 3,652 patients across 20 centers in eight countries and showed robust performance across centers, scanners, and patient ages, outperforming both expert and non-expert examiners (114). Assembling such datasets is not straightforward: linking imaging to pathology and outcome data across centers raises data-governance and privacy barriers that often keep useful data in silos (115). It is partly for this reason that external validation and clear data provenance are repeatedly cited as prerequisites for real-world adoption (84, 93).

Gynecology does, however, offer a model of what rigorous validation looks like. The IOTA program took its adnexal-mass ultrasound tools from single-center development through prospective, multi-center external validation, including assessment by examiners with varied training rather than experts alone, precisely the setting where such tools are most needed (47, 116). That trajectory, from a single-center model to prospective testing across different operators and populations, is the standard multimodal AI should be held to, and the distance most current models sit from it is a large part of why so few are in routine use.

### 6.4 Endpoint weakness

A subtler reason models fail is that they are often trained to predict the wrong thing. Many are optimized for stand-in targets that are easy to label but only loosely tied to clinical decisions, whether a scan shows response, an imaging-based guess at whether a tumor can be resected, or a segmentation that matches an expert’s outline, rather than the outcomes patients actually care about, such as living longer, avoiding complications, or receiving a better treatment. A model can hit its surrogate target exactly and still be useless if that target poorly substitutes for what matters. This is not unique to gynecology: across multimodal oncology, much of the effort still goes into biomarker discovery and risk stratification rather than the treatment decisions and actionable outcomes that would change care (83).

Encouragingly, the standard is beginning to move. Recent work is increasingly framed around decision-focused endpoints and clearer validation criteria rather than standalone model accuracy (93). The question that matters is no longer whether a model predicts well on paper, but whether its predictions improve the quality, timeliness, or safety of clinical decisions.

### 6.5 Explainability and clinical interpretability

Trust depends not only on how well a model performs but on whether clinicians can follow its reasoning. Clinician acceptance remains one of the main barriers to adopting multimodal AI, largely because many systems behave as black boxes and do not align with how clinicians actually reason (84); building trust therefore calls for explainability and an honest expression of uncertainty alongside technical accuracy. This is achievable: models that surface which factors drove each prediction have made ovarian-cancer resectability estimates something a surgeon can inspect rather than accept on faith (117). Such transparency remains the exception, and reviews across multimodal oncology note a persistent tension between the most generalizable models and the most interpretable models (93).

### 6.6 Workflow failure

The last barrier is the one model-builders most often overlook, because it appears only after the model works: the clinic itself. An accurate model still fails if its output arrives at the wrong moment, on the wrong screen, or demands attention the workflow cannot spare. Radiology, pathology, surgery, and multidisciplinary tumor boards run on tight routines, and a tool that does not fit them, whether an alert that fires too often or a result that lands after the decision it was meant to inform, is quickly ignored, the same alert fatigue clinicians already know from the electronic record (84). Real-world readiness also requires integration with PACS, DICOM viewers, and electronic medical records without introducing excessive user interaction or “click fatigue,” defined here as the cumulative workflow burden produced by repeated prompts, alerts, confirmations, or navigation steps (118).

Integration also changes how errors propagate. A confident but wrong output invites automation bias, the tendency to accept the machine’s answer without checking, which can both spread the model’s mistakes and, over time, erode the skills meant to catch them. The stakes make the risk concrete: in gynecologic oncology, an incorrect unresectability estimate could steer a patient toward neoadjuvant chemotherapy, while underestimating disease extent could distort surgical or radiotherapy planning. Even where automation clearly helps, human review stays essential; commercial deep-learning auto-contouring speeds radiotherapy planning but still requires expert supervision, because a small difference in a contour near a high-dose gradient can change the dose delivered to organs at risk (119). Responsible deployment therefore builds in explicit override pathways, audit trails, and a record of whether and how the model shaped the final decision.

Gynecologic oncology has barely begun this work: studies of how these tools behave inside real radiology, pathology, or tumor-board routines are almost nonexistent.

## 7 How gynecologic oncology AI should be validated

Clinical translation can be viewed as a stepwise progression from representative data curation and internal model development to external and prospective validation, clinical-impact evaluation, and ultimately monitored deployment (**Figure 3**).

**Figure 3:**
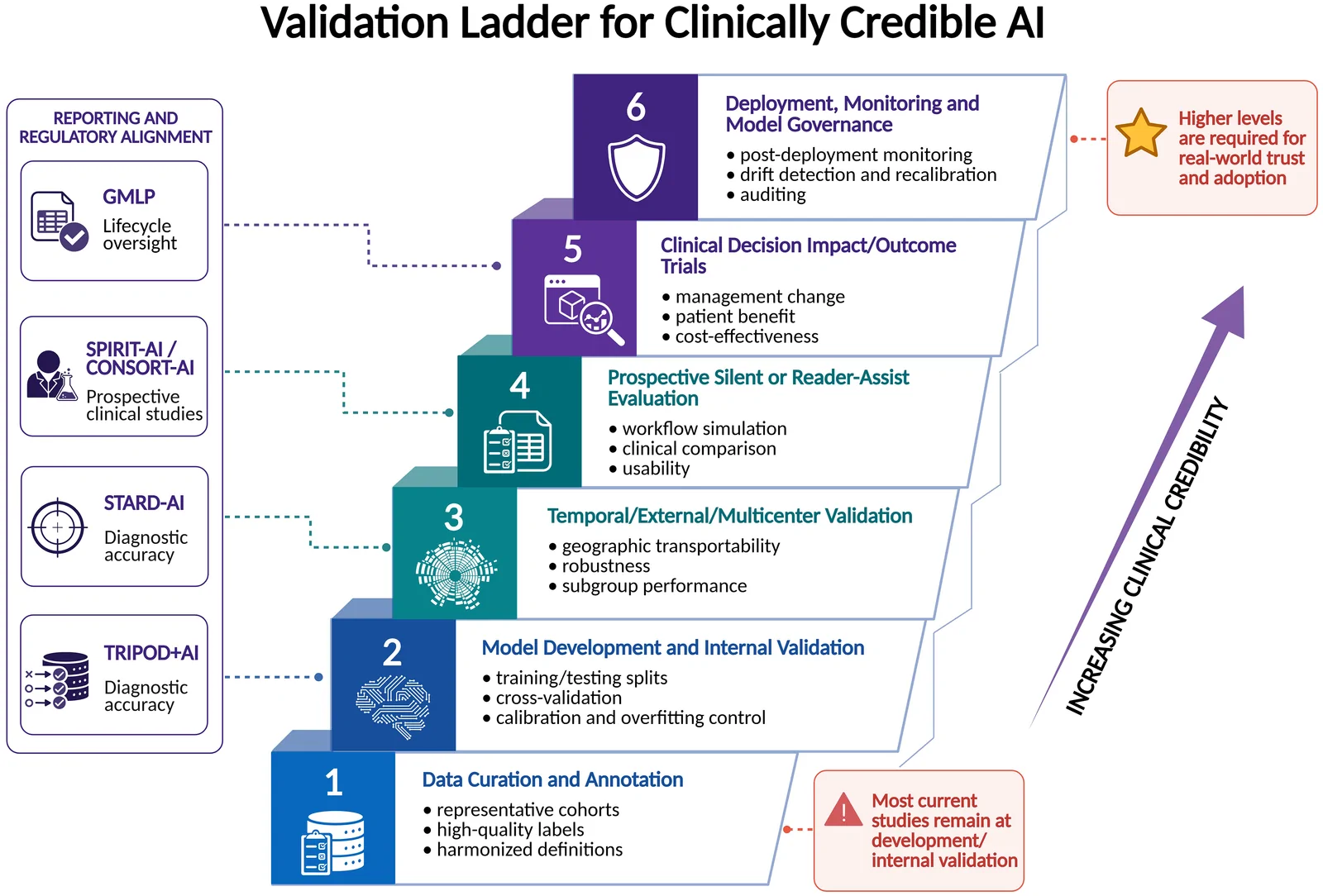
Validation ladder for clinically credible AI in gynecologic oncology. Clinical credibility increases as AI systems progress from representative data curation and internal validation to temporal, external, and multicenter testing; prospective silent or reader-assist evaluation; clinical decision-impact studies; and post-deployment monitoring and governance. Reporting and regulatory frameworks should be aligned with the corresponding stage of development, with prospective outcome evaluation and lifecycle oversight required for reliable real-world adoption. Reporting frameworks are shown according to their intended scope: TRIPOD+AI applies to the development and validation of prediction models, STARD-AI to diagnostic accuracy studies, DECIDE-AI to early live clinical evaluation, and CONSORT-AI and SPIRIT-AI to interventional trials. The stages shown should not be read as a single linear hierarchy applicable to every application. Segmentation tools, prognostic models, treatment policies, and agentic systems follow partially distinct evidence pathways, and reporting quality, analytical validation, external transportability, human-factors evaluation, clinical impact, and regulatory oversight are partially orthogonal dimensions rather than sequential rungs. Created in BioRender. Helal, B. (2026) https://BioRender.com/hypncyr.

### 7.1 External validation standards: bridging the translation gap

The transition of artificial intelligence from a technical “proof-of-concept” to a clinically deployable tool in gynecologic oncology depends on bridging the “translation gap” through standardized, multi-layered validation (2, 120). While internal validation—often performed through random split-sample or cross-validation techniques—is a standard starting point, it frequently overestimates model performance and fails to account for dataset shift (including differences in case-mix, setting, and temporal changes) encountered in real-world practice (121). Therefore, external validation should follow a targeted approach that accounts for heterogeneity across temporal changes, geographic locations, and multicenter settings, incorporating assessments of temporal transportability (stability over time), geographic transportability (performance across different institutions), and multicenter heterogeneity to ensure model robustness (121, 122).

A rigorous validation framework also requires a departure from isolated performance metrics toward comparative benchmarking against established clinical gold standards and human expertise (123). In the context of adnexal mass classification, AI models should be rigorously evaluated head-to-head against established clinical scoring systems such as the International Ovarian Tumor Analysis (IOTA) or Ovarian-Adnexal Reporting and Data System (O-RADS) to determine whether they provide meaningful incremental diagnostic gain (124). Furthermore, validation studies should distinguish between “expert” and “non-expert” settings; a model that merely matches the performance of a generalist may provide limited value in a tertiary oncology center compared to one that demonstrates non-inferiority to a sub-specialized gynecologic oncologist (4). Ultimately, the reporting of these validation efforts must be transparent regarding annotation inconsistency and domain shift, ensuring that the final output is technically auditable and clinically reliable across diverse global care settings (125).

### 7.2 Prospective validation pathway

The first stage of prospective validation involves silent-reader studies, where the AI algorithm processes live clinical data, such as real-time pelvic ultrasound or newly acquired PET/CT scans, in the background without the results being revealed to the treating physician (126). This “shadowing” methodology is critical for establishing the model’s real-world baseline, as it exposes the algorithm to “unfiltered” data, including motion artifacts, sub-optimal contrast timing, and operator-dependent variations that are typically removed from retrospective training sets (127).

Recent feasibility studies in gynecologic oncology have applied this approach to compare AI-generated recommendations with Multidisciplinary Tumor Board (MDT) decisions. These studies typically demonstrate high concordance in straightforward cases, yet also highlight that AI often struggles to match the nuanced clinical reasoning required for complex, multi-modal treatment pathways (4).

Once a baseline is established, reader-assist (or human-AI team) studies are employed to evaluate the synergistic effect of the “human-AI” team. Rather than testing the AI as an autonomous agent, these trials measure whether a clinician’s diagnostic accuracy, particularly that of a non-expert generalist, improves significantly when augmented by AI outputs (128).

In the context of adnexal mass characterization, such studies frequently benchmark enhanced multimodal approaches against established human frameworks like O-RADS US v2022 (129). Evidence suggests that AI-assisted ultrasound interpretation can provide an incremental diagnostic gain over sonographers, with higher sensitivity and preserved high specificity that may help avoid unnecessary surgical intervention in benign case (130). The true litmus test of a clinical AI tool is its ability to influence management decisions that ultimately benefit the patient. In advanced ovarian cancer, this includes improving prediction of complete cytoreduction to better guide the choice between primary debulking surgery (PDS) and neoadjuvant chemotherapy (NACT) (131). However, recent research underscores a persistent “translation gap”: while AI systems can accurately recognize clinical abnormalities, they often diverge from Multidisciplinary Tumor Board (MDT) recommendations in complex or atypical cases (132).

These discrepancies highlight that AI is currently best suited as a decision-support aid rather than a replacement for human judgment in high-stakes oncology settings (133).

Finally, the “gold standard” for clinical integration is the prospective outcome study, which transitions the primary endpoint from radiological surrogate labels (e.g., AUC or sensitivity) to meaningful patient outcomes. These studies evaluate whether the implementation of an AI tool leads to improved Progression-Free Survival (PFS), higher rates of R0 surgical resections, or a reduction in treatment-related toxicities (93). While retrospective nomograms have long attempted to predict survival in ovarian cancer based on preoperative variables, the next generation of gynecologic oncology AI must demonstrate its ability to dynamically optimize these outcomes in real time (2). Achieving this level of validation requires massive, multi-institutional collaborations to ensure the AI’s impact is consistent across diverse global care settings and patient populations (134).

### 7.3 Clinically meaningful performance reporting

While the Area Under the Receiver Operating Characteristic Curve (AUC) remains the most ubiquitous metric in medical AI, it is essentially a measure of discrimination—the model’s ability to rank a patient with a disease higher than one without it (135). In a clinical vacuum, a high AUC is impressive; however, in the management of gynecological malignancies, discrimination alone is insufficient. A model may have an AUC of 0.95 yet be clinically dangerous if it is poorly calibrated (136). For instance, overestimation of lymph node metastasis risk in patients with endometrial cancer can lead to unnecessary lymphadenectomies with associated morbidity. Preoperative prediction models therefore require careful evaluation of discrimination, calibration, and clinical utility to support safe de-escalation of surgical staging (137).

Calibration refers to the agreement between the predicted probability of an event and its observed frequency. In oncology, where treatment decisions are increasingly personalized based on risk thresholds, a miscalibrated model can be catastrophic. To avoid the common pitfall of ignoring calibration, researchers must utilize reliability curves and the Brier Score (138). In the context of O-RADS or IOTA comparisons, a well-calibrated model lets the oncologist discuss “real” percentages of risk with the patient, supporting shared decision-making rather than relying on a “black-box” binary output (138).

Assess calibration in the population in which the model is intended to be used, and do not infer it from discrimination alone. Appropriate assessment may include calibration-in-the-large, calibration intercept and slope, flexible calibration plots, and, for time-to-event models, calibration at clinically relevant time horizons. Because prevalence and case mix can change across institutions, recalibration may be necessary after external transport even when the AUC remains relatively stable.

Decision-curve analysis provides an important complementary assessment of decision-analytic utility by estimating net benefit across clinically relevant risk thresholds. Unlike discrimination alone, it incorporates the relative consequences of false-positive and false-negative decisions and can identify ranges in which use of a model may provide greater net benefit than alternative strategies such as treating all or treating none. However, decision-curve analysis does not demonstrate that implementation improves patient outcomes, workflow safety, effectiveness, or cost-effectiveness. These questions require subsequent human–AI evaluation and prospective clinical-impact studies (138). A summary of these key performance metrics and their clinical targets is provided in **Table 1**. Unlike sensitivity and specificity, which treat false positives and false negatives as equal, DCA acknowledges that the “cost” of a missed cancer diagnosis is significantly higher than the “cost” of an unnecessary intervention. By plotting the Net Benefit of an AI model against the “treat-all” or “treat-none” strategies, clinicians can visualize the exact range of risk thresholds where the AI provides a superior clinical outcome (138).

**Table 1:** Key performance metrics for the clinical validation of Al models in gynecologic oncology, including the clinical questions answered and target values.

Metric	Clinical question answered	Target for gynecologic oncology
AUC	Can the AI tell the difference between a tumor and a cyst?	Task-specific; report confidence intervals and performance at prespecified clinically meaningful operating thresholds relative to the relevant comparator
Calibration	Does a “20% risk” prediction actually mean 20 out of 100?	Slope near 1.0; Intercept near 0
Net Benefit	Does using this AI lead to better outcomes than the status quo?	Positive NB across all relevant thresholds
Brier Score	How “off” are the model’s individual predictions?	As close to 0 as possible

A critical, often overlooked component of rigorous AI validation is Uncertainty Quantification (UQ). In high-stakes environments such as radiotherapy planning for cervical cancer or predicting platinum sensitivity in ovarian cancer, a model must “know what it doesn’t know” (139). Methods such as Monte Carlo Dropout and Deep Ensembles enable models to output a confidence interval or uncertainty score alongside each prediction. When the model encounters a “wildcard” case, for example, a rare uterine sarcoma subtype underrepresented in the training data, it should flag this as a high-uncertainty case, triggering mandatory expert human review. This approach helps prevent dangerous forced-choice errors, reduces clinician alert fatigue, and preserves trust in the AI system (140).

A critical pillar of rigorous validation is the comprehensive auditing of subgroup performance (e.g., by analysis method or validation setting) to ensure that high aggregate accuracy does not obscure hidden performance differences. In gynecological oncology, models trained primarily on data from high-resource or single-center settings often show performance decay on external validation cohorts, underscoring the need for proactive evaluation across diverse populations, disease phenotypes, and clinical settings to ensure reliable real-world performance (130). Furthermore, technical fairness must be established by demonstrating that the algorithm remains robust against domain shift, maintaining consistent predictive power across varying scanner manufacturers, image acquisition protocols, and the inherent operator-dependence characteristic of pelvic ultrasonography (141). Beyond demographic and technical variables, models must be scrutinized for their performance across clinically distinct subgroups, such as rare histologic subtypes or specific molecular classifications—including POLE-mutated versus p53-abnormal endometrial cancers—where small sample sizes often lead to systematic under-performance in generalized models (142).

### 7.4 Reporting and regulatory frameworks

The path toward clinical implementation is paved with standardized reporting, which serves as the primary antidote to the “black-box” reporting often seen in early-stage AI research. To ensure transparency and reproducibility, all AI-centered research in gynecologic oncology must adhere to evolving reporting guidelines, specifically the TRIPOD+AI statement for predictive modeling (143). These frameworks demand the disclosure of granular technical details—such as handling of missing data, exact multivariable modeling techniques, and calibration metrics—to transition from isolated pilot studies to technically auditable medical evidence (143).

For diagnostic accuracy studies, the STARD-AI reporting guideline provides a dedicated standard designed to improve completeness and transparency of AI-centered diagnostic test accuracy studies and to better address AI-specific challenges such as data preprocessing, model development, external validation, fairness, and explainability, thereby mitigating risk of bias and enhancing applicability to diverse clinical workflows (144).

Furthermore, when AI tools transition from passive observation to active intervention, such as directly influencing surgical or systemic treatment selection- adherence to the CONSORT-AI and SPIRIT-AI reporting guidelines becomes essential (145).

### 7.5 Defining “real-world readiness”

Defining “real-world readiness” in gynecologic oncology requires moving beyond isolated performance metrics to address the broader challenges of interoperability, auditability, and long-term stability (146). A model is only clinically ready if it demonstrates technical reproducibility across international, multicenter settings, maintaining its predictive power despite the inherent “noise” of site-specific technical protocols and varying patient demographics (146).

Crucially, real-world readiness hinges on interoperability, specifically the ability of the AI tool to integrate seamlessly into existing PACS, DICOM viewers, and EMR systems without introducing significant “click fatigue” or disrupting the fast-paced routines of multidisciplinary tumor boards. To maintain clinician trust, systems must remain fully auditable, leveraging Explainable AI (XAI) techniques to provide a transparent rationale for recommendations, enabling oncologists to verify that the model is identifying genuine biological features rather than technical artifacts (147).

Once deployed, institutions must establish rigorous post-deployment monitoring protocols to detect model decay or performance shifts in real time. A robust framework for local retraining and update governance is equally critical; this enables fine-tuning to local populations while mitigating risks such as catastrophic forgetting or site-specific biases that could compromise patient safety (148). When governed by strict validation and oversight, such continual learning approaches are essential for safe, equitable, and sustainable long-term deployment of AI in gynecologic oncology.

## 8 Ethical, regulatory, and implementation challenges

### 8.1 Data governance and privacy

AI models in gynecologic oncology are increasingly moving from single-image classification toward multimodal prediction systems that combine ultrasound, MRI, CT, PET/CT, digital pathology, serum biomarkers, clinicopathologic variables, molecular subtype, treatment history, and longitudinal outcomes. This shift creates a direct governance problem: the models most likely to support treatment decisions require datasets that are larger, more diverse, and more longitudinal than any single center can usually provide.

In gynecologic oncology, de-identification cannot be viewed as a simple removal of obvious identifiers such as names and hospital numbers. Imaging files often contain protected metadata, burned-in annotations, acquisition dates, device identifiers, and institution-specific information. Moreover, multimodal linkage to histology, genomic data, recurrence, survival, and treatment response substantially increases re-identification risks (149, 150). This challenge is amplified for rare gynecologic tumors and uncommon molecular subtypes, where small case numbers heighten identifiability even after standard anonymization. Effective privacy-preserving AI therefore requires not only robust technical de-identification of DICOM data but also comprehensive governance frameworks addressing linkage keys, data-access permissions, audit trails, cybersecurity, and secondary data use (149, 150).

However, federated learning should be presented as a partial solution rather than a complete answer to data governance. While it reduces raw data movement, it does not automatically solve bias, poor annotation quality, institutional imbalance, cybersecurity risks, or unequal technical infrastructure (151, 152). Federated models can still inherit non-representative sampling, scanner-specific features, local treatment practices, and center-specific outcome definitions. In gynecologic oncology, this matters because model performance may depend heavily on ultrasound expertise, MRI protocol quality, access to molecular testing, surgical referral pathways, and follow-up completeness. Thus, privacy-preserving collaboration must be paired with harmonized annotation standards, common outcome definitions, transparent data-use agreements, and prospective monitoring of model behavior across sites.

Consent and stewardship are equally important. Many AI studies rely on retrospectively collected clinical data, yet patients may not expect that routine imaging, pathology, or molecular results will later be used to develop predictive algorithms, particularly if commercial partners are involved. For high-stakes treatment-support models, governance should move beyond one-time de-identification toward lifecycle stewardship: clear consent or waiver frameworks, patient-level protections, institutional accountability, documentation of intended secondary use, and mechanisms for monitoring model updates after deployment (149, 150). For gynecologic oncology AI to become clinically trustworthy, data governance must therefore be treated as part of model quality, not as an administrative step after model development.

### 8.2 Algorithmic bias and equity

Algorithmic bias and limited generalizability in gynecologic oncology AI models can arise from dataset composition, image acquisition variability across centers and devices, and annotation practices (114, 153). Many current models are developed using retrospective clinical cohorts assembled from routinely acquired data at tertiary referral centers, where patient populations, imaging protocols, equipment, and access to molecular testing differ substantially from those in community or lower-resource settings (55, 114, 153).

Recent multicenter ovarian ultrasound studies illustrate both the promise and the fragility of AI generalizability. Christiansen et al. validated a transformer-based model for ovarian cancer detection across 20 centers in eight countries, using 17,119 ultrasound images from 3,652 patients. Their results demonstrated that large, heterogeneous training datasets can substantially improve cross-site robustness (114). Similarly, Dai et al. developed the OvaMTA deep learning pipeline using multicenter ultrasound data from 21 hospitals and externally validated the system in additional centers, again emphasizing that external testing is essential for clinically credible performance claims (153). However, these studies also indirectly highlight the scale of the bias problem: differences in sonographer expertise, ultrasound hardware, acquisition protocols, referral populations, and prevalence of malignant disease can all influence model behavior. Therefore, strong internal performance does not guarantee equitable deployment across different clinical environments. This issue is particularly relevant for ovarian and endometrial cancers, which exhibit considerable heterogeneity in imaging characteristics, histopathologic features, and molecular subtypes across institutions and geographic regions.

Bias is not only a technical problem; it can directly influence access to diagnosis and treatment. Both Christiansen et al. and Dai et al. developed their models primarily on data from tertiary referral centers and multicenter hospital networks. While these models generalized well across centers, ultrasound systems, and patient subgroups in their validation cohorts, the authors acknowledge important limitations in transferability to settings with lower imaging quality, less experienced sonographers, or different clinical workflows and referral patterns. This creates a risk that AI systems optimized on data from well-resourced tertiary environments may offer less reliable support when deployed in more diverse or lower-resource clinical settings (154).

Algorithmic inequity is especially concerning because gynecologic malignancies are globally uneven diseases. Cervical cancer provides the clearest example: disease burden remains highest in low- and middle-income regions where access to screening, HPV testing, pathology, subspecialty imaging, and oncologic treatment is often limited. Yet most AI development datasets continue to originate from high-resource environments in North America, Europe, and parts of East Asia (12, 13). This creates a structural paradox: populations with the greatest unmet gynecologic oncology burden are often underrepresented during model development. Consequently, AI systems optimized in highly resourced tertiary centers may underperform in rural hospitals, lower-field imaging systems, limited-resource pathology settings, or fragmented referral networks.

At the same time, AI also carries genuine potential to expand access if implemented appropriately. A large real-world cervical cancer screening program in China integrated AI-assisted and cloud-based cytology assessment across a population of approximately 1.7 million women, with rural participants accounting for more than two-thirds of screened individuals. The program demonstrated substantial improvements in screening efficiency and workload reduction compared with conventional manual interpretation (155). However, such studies should not be interpreted as evidence that AI automatically improves equity. Screening algorithms remain dependent on sample quality, confirmatory pathology, referral systems, treatment availability, patient follow-up, and local governance structures. In practice, AI may alleviate one bottleneck while leaving broader structural disparities unchanged.

Equity should therefore be treated as a core validation endpoint, not a secondary ethical discussion. Future gynecologic oncology AI studies should predefine subgroup analyses, evaluate performance across external institutions and resource settings, report missingness and exclusion patterns, and test whether AI recommendations change management differently across patient groups. Ultimately, an AI model should not be considered clinically mature unless it performs reliably in the populations most likely to depend on it, including non-tertiary, rural, and lower-resource settings.

### 8.3 Accountability and clinician oversight

Accountability becomes particularly complex when AI supports treatment decisions rather than image classification alone. In gynecologic oncology, an AI output may influence whether a patient is referred for primary debulking surgery, neoadjuvant chemotherapy, nodal assessment, adaptive radiotherapy, or intensified surveillance. When such recommendations are wrong, responsibility is distributed across the clinician, institution, software vendor, data curator, and model developer. This is why early clinical evaluation frameworks such as DECIDE-AI emphasize not only model performance, but also human factors, intended use, clinical workflow integration, safety monitoring, and the interaction between clinicians and AI outputs (79).

Clinician oversight should therefore be active, not symbolic. The phrase “human-in-the-loop” often reassures readers that the physician retains final decision-making authority, but this is insufficient if the clinician lacks access to model uncertainty, training context, subgroup performance, or reasons for recommendations. Direct evidence on over-reliance is scarce within gynecologic oncology, and the available signals are indirect. A cross-sectional comparison of medication dose calculation among nurses, nursing students, and generative AI models found that generative models were regarded as an additional verification layer rather than an independent authority, and that the error-prevention strategies practitioners themselves prioritized centered on independent double-checking. This is indirect evidence, drawn from a different clinical task and a different workforce, and it should not be read as gynecologic validation; it nonetheless supports the general principle that an algorithmic output is safe only where an independent check remains in place (156).

The need for oversight is especially clear in radiotherapy, where AI has moved closer to operational use than in many other gynecologic oncology domains. Researchers have developed deep learning models for per-fraction segmentation of gross tumor volume and organs at risk in adaptive radiotherapy for cervical cancer (157). Prospective evaluation of automated contouring for CT-based gynecologic brachytherapy showed that auto-contouring can streamline workflow and significantly reduce time to contour approval and overall planning time. However, such systems still require clinician review, as small contouring differences (particularly in small bowel and sigmoid) may affect dose-volume constraints for organs at risk, even when dosimetric impact is minimal (158). Similarly, a global evaluation of an AI-based radiotherapy planning assistant (Radiation Planning Assistant) demonstrated high clinical acceptability for cervical cancer, with normal tissue autocontours acceptable as-is in 92%, clinical target volumes in 83%, and VMAT plans in 99%. However, the frequent need for minor edits underscores that clinician verification remains essential (159). These studies support AI as workflow augmentation, not autonomous radiotherapy planning.

Interpretability is another major requirement for accountable oversight. Oncology decisions often involve competing risks, patient preferences, comorbidity, fertility goals, surgical expertise, and institutional resources; therefore, a black-box prediction is rarely sufficient for management. Lu et al. argued that interpretable machine learning predictions are especially important in oncology because clinical action requires understanding not only the predicted risk, but also the patient-specific drivers of that risk (160). However, explainability should not be reduced to visually attractive heatmaps or saliency maps. Many *post-hoc* explanations are unstable, non-causal, or hard to translate into management decisions. For gynecologic oncology, useful interpretability should answer clinically actionable questions: which imaging region, clinical variable, molecular feature, or treatment factor shifted the recommendation, and would the recommendation change if that variable were absent or uncertain?

Finally, clinician oversight must extend to patient communication. When AI contributes to high-stakes treatment planning, patients should understand that the tool is supportive rather than determinative and that the clinical team retains final responsibility. This transparency is especially important when AI recommendations influence fertility-sparing options, radical surgery, radiotherapy dose, or systemic therapy intensity.

### 8.4 Regulatory approval and post-market surveillance

Regulating medical artificial intelligence differs fundamentally from regulating conventional software because many AI systems are probabilistic, data-dependent, and potentially adaptive after deployment (161). Similarly, the European Union Artificial Intelligence Act classifies many clinical AI systems as high-risk technologies and mandates requirements for transparency, documentation, human oversight, and post-market monitoring. International guidance from the International Medical Device Regulators Forum (IMDRF) has similarly emphasized Good Machine Learning Practice (GMLP) principles to promote safe and effective clinical AI deployment (162). Consequently, regulatory approval is increasingly shifting from a single premarket evaluation toward a total product lifecycle approach that incorporates predeployment validation, post-market monitoring, update governance, and continuous safety assessment (161).

Current regulatory frameworks increasingly classify AI-based oncology systems under the broader category of Software as a Medical Device (161). Despite rapid regulatory progress, important uncertainties remain regarding explainability requirements, acceptable model updating, liability attribution, and thresholds for reapproval after algorithmic modification.

A central regulatory challenge is the distinction between “locked” and adaptive algorithms. Most currently deployed medical AI systems remain effectively locked models whose outputs do not substantially change after deployment (163). In contrast, continuously learning systems may evolve as new clinical data become available, creating concerns regarding reproducibility, calibration drift, and safety oversight (162). To address this issue, the FDA introduced the concept of Predetermined Change Control Plans, which prospectively define permitted post-deployment modifications and specify how these updates will be validated and monitored (162).This framework acknowledges that clinically useful AI systems may require controlled evolution rather than permanent immutability, although implementation remains challenging.

Performance drift after deployment represents another major challenge. AI systems validated in one institutional or temporal setting may deteriorate when exposed to scanner upgrades, altered acquisition protocols, evolving treatment pathways, changing referral patterns, or shifts in disease prevalence (27). In gynecologic oncology, these effects may be amplified by differences in ultrasound expertise, MRI vendor protocols, availability of molecular testing, and surgical selection practices between institutions.

Post-market surveillance is consequently becoming a central component of medical AI governance. Emerging frameworks increasingly recommend continuous monitoring of calibration, subgroup performance, dataset drift, clinically significant errors, and real-world safety outcomes after deployment (164). Recent governance proposals such as AEGIS further illustrate how adaptive AI systems may eventually require automated monitoring infrastructures capable of detecting drift, triggering clinical review, and restricting deployment when predefined safety thresholds are exceeded (165). Although many of these systems remain conceptual, they reflect an important transition from static regulatory approval toward dynamic operational oversight.

## 9 A roadmap for the next generation of reviews and studies

### 9.1 Priority research questions

The current AI landscape in gynecologic oncology must evaluate which applications—particularly ovarian ultrasound characterization, endometrial MRI-based risk assessment, and cervical cancer radiotherapy planning are realistically closest to routine workflow deployment. Furthermore, the field needs greater emphasis on identifying clinically meaningful endpoints that directly influence treatment planning, including chemotherapy response, molecular risk stratification, recurrence risk, and treatment toxicity. Prioritizing these decision-focused endpoints will shift the field from proof-of-concept models toward reproducible, clinically integrated decision-support systems.

### 9.2 What multicenter collaborations should look like

Future multicenter collaborations must address several foundational methodological challenges to improve reproducibility and clinical applicability. Harmonized imaging acquisition protocols are crucial for minimizing variability across scanner manufacturers, reconstruction algorithms, and institution-specific imaging parameters (166). Oncology initiatives such as DATECAN illustrate the importance of prespecifying standardized time-to-event endpoint definitions; however, select or adapt disease-specific endpoint definitions for gynecologic oncology rather than transferring them uncritically across tumor types. Publicly accessible resources, including imaging repositories such as The Cancer Imaging Archive and molecular and clinicogenomic resources such as The Cancer Genome Atlas, can support benchmarking, multimodal linkage, hypothesis generation, and external model evaluation. However, available gynecologic cohorts remain limited by incomplete pairing of imaging, pathology, molecular, treatment, and longitudinal outcome data (167).

### 9.3 Future technologies

Several emerging technologies show promise for advancing AI in gynecologic oncology. Foundation models trained on large-scale medical imaging datasets could reduce dependence on highly disease-specific labeled cohorts and improve adaptability across imaging tasks (168)). Additionally, weakly supervised and self-supervised learning approaches may improve efficiency by extracting informative representations from partially labeled or unlabeled datasets, offering an especially important advantage in low-resource settings. Beyond prediction alone, causal AI may eventually enable systems to estimate treatment effects rather than simply simulate outcomes (169). For example, such approaches could potentially predict how a specific chemotherapy regimen might influence progression-free survival in an individual patient. However, such methods remain highly experimental and will require rigorous prospective validation before clinical implementation. Similarly, digital twins represent an emerging approach in oncology. These patient-specific computational models simulate tumor behavior, disease progression, and treatment response by integrating multimodal data into a continuously updated “virtual patient” (170). In principle, this framework could allow in silico testing of different therapeutic strategies to support more individualized clinical decision-making. However, in this context, digital twins remain largely conceptual, constrained by data-integration challenges and the lack of prospective validation. Collectively, these emerging approaches signal a transition toward more generalizable, data-efficient, and causally grounded AI systems, with the potential to meaningfully enhance personalized decision-making in gynecologic oncology pending further validation.

### 9.4 The realistic near-term role of AI

The most viable applications of AI in gynecologic oncology are currently in clinical decision support rather than autonomous treatment selection. These systems are most likely to function as augmentation tools embedded within multidisciplinary workflows. In radiology, AI-assisted detection of nodal disease and subtle imaging abnormalities may improve diagnostic consistency and reduce cognitive workload. Meanwhile, in radiation oncology, automated organ-at-risk segmentation can streamline adaptive treatment planning. In pathology, quantitative assessment of features such as tumor-infiltrating lymphocytes and necrosis may enhance reproducibility and reduce interobserver variability. Within multidisciplinary tumor boards, AI-generated integration of imaging, pathology, molecular, and clinical data may support more standardized and guideline-concordant decision-making.

## 10 Conclusion

AI in gynecologic oncology has reached a transition point from isolated image classification toward systems that integrate imaging, pathology, molecular biomarkers, and clinical information. The strongest near-term opportunities are likely to remain disease- and workflow-specific: ultrasound-supported adnexal mass triage and surgical referral in ovarian cancer, integration of MRI with molecular risk classification in endometrial cancer, and segmentation and treatment-planning support in cervical radiotherapy. These examples also illustrate why a single model-development strategy is unlikely to fit the entire field, because the relevant biology, endpoints, data quality, and clinical consequences differ substantially among gynecologic malignancies.

The principal challenge is therefore not simply achieving higher retrospective accuracy, but demonstrating that an AI system adds clinically meaningful information beyond established imaging, pathology, molecular assays, and multidisciplinary expertise. Multimodal models will be most valuable where complementary data genuinely alter risk estimates or management, whereas well-validated unimodal systems may remain preferable for narrowly defined tasks. For survival and treatment-selection applications, future studies must move beyond prognostic association toward externally validated and calibrated models linked to prespecified clinical actions and, ultimately, prospective evidence of benefit. In the foreseeable future, AI’s most credible role is clinician-supervised decision support embedded within gynecologic oncology teams, with success measured by improvements in consistency, efficiency, safety, and patient-important outcomes rather than predictive performance alone.
